# Prevalence of myopia and vision impairment in school students in Eastern China

**DOI:** 10.1186/s12886-019-1281-0

**Published:** 2020-01-02

**Authors:** Jianyong Wang, Gui-shuang Ying, Xiaojin Fu, Ronghua Zhang, Jia Meng, Fang Gu, Juanjuan Li

**Affiliations:** 1 Department of Ophthalmology, The First Affiliated Hospital, College of Medicine, Zhejiang University, 79 Qingchun Road, Hangzhou, 310003 People’s Republic of China; 2 Center for Preventive Ophthalmology and Biostatistics, Department of Ophthalmology, Perelman School of Medicine, University of Pennsylvania, Philadelphia, PA USA; 3 Department of Ophthalmology, Central Hospital of Yiwu City, Yiwu, Zhejiang Province People’s Republic of China; 4 Center for Disease Prevention and Control, Hangzhou, Zhejiang Province People’s Republic of China

**Keywords:** Myopia, Vision impairment, School students

## Abstract

**Background:**

Prevention of myopia has become a public health priority in China. This study is to investigate the prevalence of myopia and vision impairment, and their associated factors in school students in eastern China.

**Method:**

In this cross-sectional school-based study of 4801 students from 16 schools ranging from kindergarten to high school, students underwent refraction using non-cycloplegic autorefractor and visual acuity testing using logMAR chart with tumbling E. Myopia was defined as spherical equivalent (SPHE) ≤ − 0.5 diopter (D) and uncorrected visual acuity (UCVA) 20/25 or worse. High myopia was defined as SPHE ≤ − 6.0 D and UCVA 20/25 or worse. Vision impairment was defined as UCVA 20/40 or worse. Logistic regression models were used to determine factors associated with myopia and vision impairment.

**Results:**

Among 4801 children (55% male) with mean age (standard deviation) 12.3 (3.8) years, 3030 (63.1, 95% CI: 61.7–64.5%) had myopia, 452 (9.4, 95% CI: 8.6–10.3%) had high myopia, and 2644 (55.1, 95% CI, 53.7–56.5%) had vision impairment. The prevalence rate of myopia increased with grade in a non-linear manner, 12% in kindergarten, 32% in grade 2, 69% in grade 5, and approximately 90% by grade 10 or above. The prevalence rate of high myopia was relatively low in grade 4 or below (< 1.5%), 4–7% in grade 5 to 7, 13–15% in grade 8–9, and > 20% in grade 10 to 12. The prevalence rate of vision impairment was 4% in kindergarten, 37% in elementary school, 77% in middle school and 87% in high school students. Higher grade (*p* < 0.0001), female (*p* < 0.0001) and higher school workload (*p* = 0.007) were independently associated with higher prevalence rates of myopia and vision impairment, while higher grade (*p* < 0.0001) and higher school workload (*p* < 0.0001) were independently associated with higher prevalence of high myopia.

**Conclusion:**

Prevalence of myopia and vision impairment was high among Chinese school students and increased with grade in a non-linear manner, reaching alarming high in high school students accompanied by high prevalence of high myopia. Increasing study burden on school students at younger age plays an important role on the higher prevalence rate of myopia and vision impairment.

## Background

The prevalence of myopia has been increasing and reached alarming high rate in middle or high school students (> 80%) in East Asia [1, 2]. Myopia is projected to affect 50% of the world population by 2050 [3], and is recognized as a major twenty-first century public health problem globally [4, 5]. As a significant cause of vision loss, myopia is associated with ocular diseases including glaucoma and retinal detachment. Prevention of myopia has become an important public health priority in China.

Both genetic and environment factors play important roles in myopia development and progression*,* although their exact mechanisms are not yet known. Epidemiological studies have demonstrated that many factors are associated with higher rate of myopia including increasing age, larger amount of near work, family history, while more hours of outdoor activities are associated with lower risk of myopia [6–9]. The socioeconomic status and education burden on students have been reported to be associated with myopia prevalence [10, 11]. Although it is well-known that the myopia prevalence is high in China, studies suggested that there is high variation in the myopia prevalence across geographical regions of China, likely due to the differences in socioeconomic status and the education intensity of students [11]. In recent years, China has been undergoing dramatic economic development, accompanied by the increasing education pressure on the young children. These changes may have substantial impact on the myopia prevalence. The purpose of this study is to evaluate the prevalence of myopia in a high socio-economic region in East China, where educational pressure on students from schooling systems and parental expectation on academic achievements are higher than most regions of eastern China.

## Methods

This is a cross-sectional school-based study of myopia prevalence conducted in May 2019 in YiWu, a county-level city of about 1.2 million people in central Zhejiang province, P. R. China. The city is famous for its small commodity trade and vibrant market (thus called International Trade City). Yiwu is well-developed and ranked as one of top 10 richest counties in P.R. China.

For this study, a random sample of 18 schools including kindergarten, elementary school (grade 1 to 6), middle school (grade 7–9) and high school (grade 10–12) were selected. Among the selected schools, random sample of classes from each grade were selected and all students from the selected classes were invited to participate the study to reach at least 80 students from each grade of the selected schools.

The information on student name, date of birth, gender and grade were obtained from each selected classes of the school. The information on school workload was obtained from school to classify school as low, moderate and high school workload based on the number of hours in class during school day, homework assignments and frequency of tests at school. All participants underwent eye examination following the standard study protocol by the trained eye-care professionals (optometrists or ophthalmologists) for common ocular diseases, tests for the distance visual acuity using retro-illuminated logMAR chart with tumbling-E optotypes followed by measuring for refractive error using table-mounted NIDEK noncycloplegic autorefractor (Model: AR-1 s, Japan). Three readings of refractive error were taken from each eye and their average was entered for analysis. If the difference between any of two readings from an eye was greater than 0.5 diopters, refractive error for that eye was re-taken.

Before test of visual acuity, students were asked whether they wear glasses, contact lens or orthokeratology contact lens. For the students not wearing glasses, the uncorrected visual acuity (UCVA) was tested for each eye. For those wearing glasses, visual acuity was first measured without correction, then was measured with best correction at 30 min after removing the glasses. Students who were found having ocular diseases (pediatric cataract, glaucoma, optic neuropathy) or ocular injuries other than the significant refractive error were not eligible for the study.

For quality control, autorefractors were calibrated every day for measuring refractive error. Approximately 5% of students were randomly chosen to repeat the test of refraction. The data were double entered into excel sheets and the differences were resolved by checking with the original paper record.

The study was approved by the Institutional Review Board. Written informed consent was obtained from legal parent/guardian for participants less than 18 years old, and from participants directly for those 18 years or older.

The study was designed to provide precise estimate of prevalence rate of myopia and hyperopia. The sample size of 4800 students in this study provides half length of 95% confidence interval of 1.4% for myopia and 0.8% for high hyperopia assuming the prevalence rate of 60% for myopia and 9% for high hyperopia.

### Statistical analysis

Spherical equivalent for each eye was calculated as sphere plus half of the cylinder. Because the correlation between left eye and right eye is high for both spherical equivalent and visual acuity (Pearson correlation coefficient = 0.89 for both spherical equivalent and uncorrected visual acuity), the spherical equivalent from the more myopic eye and the uncorrected visual acuity from eye with worse visual acuity was used for analysis. Since the refractive error was measured using non-cycloplegic autorefractor, which tended to over-measure the myopic magnitude, particularly in young children [12, 13], we defined myopia using combination of spherical equivalent and UCVA. Myopia was defined as spherical equivalent ≤ − 0.5 diopter and UCVA 20/25 or worse. High myopia was defined as spherical equivalent ≤ − 6.0 diopters and UCVA 20/25 or worse. Including the UCVA into the definition of myopia was found to improve the accuracy of myopia using non-cycloplegic autorefractor refractive error [14]. Vision impairment was defined as UCVA 20/40 or worse in either eye.

Comparisons of spherical equivalent across levels of each risk factors were performed using analysis of variances, and comparisons of prevalence rates of myopia, high myopia and vision impairment across levels of each risk factor were performed using the chi-square test. To evaluate the independent association of the each risk factors (grade, gender, school location, school workload) with refractive error and prevalence of myopia and vision impairment, multivariate linear regression model was used for refractive error, and multivariate logistic regression models were used for myopia and vision impairment. In the multivariate regression models, the grade instead of age was used, because age and grade was highly correlated (Pearson correlation coefficient of 0.99) and grade was slightly more predictive of myopia and vision impairment than age. All the statistical analyses were performed in SAS v9.4 (SAS Institute Inc., Cary, NC) and two-sided *p* < 0.05 was considered to be statistically significant.

## Results

Total of 4801 school-aged students from 16 schools of 8 towns in YiWu were enrolled into study. The student grade ranged from Kindergarten to grade 12 with number of students ranging from 331 to 403 in each grade. The mean (SD) of age was 12.3 (3.8) years ranging from 5 to 20 years, with 2647 (55.1%) male, and 2691 (56.1%) were from urban schools. Majority of students (80.0%) were from moderate workload schools, and 657 (13.7%) were from high workload schools (Table 1). Among all students, 3030 (63.1, 95% CI: 61.7–64.5%) had myopia, and 452 (9.4, 95% CI: 8.6–10.3%) had high myopia. The UCVA worse than 20/40 occurred in 2644 (55.1, 95% CI: 95% CI: 53.7–56.5%) students, including 364 (7.6%) students with UCVA 20/200 or worse.

**Table 1:** Characteristics of Study Participants (*N* = 4801)

Demographics	
Age in Years	n (%)
5	1 (0.02%)
6	292 (6.1%)
7	345 (7.2%)
8	359 (7.5%)
9	369 (7.7%)
10	360 (7.5%)
11	385 (8.0%)
12	353 (7.3%)
13	374 (7.8%)
14	365 (7.6%)
15	371 (7.7%)
16	361 (7.5%)
17	370 (7.7%)
18	370 (7.7%)
19	121 (2.5%)
20	5 (0.1%)
Mean (SD)	12.3 (3.8)
Gender	
Male	2647 (55.1%)
Female	2154 (44.9%)
Grade	
Kindergarten	381 (7.9%)
Grade 1	331 (6.9%)
Grade 2	366 (7.6%)
Grade 3	371 (7.7%)
Grade 4	367 (7.6%)
Grade 5	376 (7.8%)
Grade 6	368 (7.7%)
Grade 7	376 (7.8%)
Grade 8	360 (7.5%)
Grade 9	346 (7.2%)
Grade 10	383 (8.0%)
Grade 11	373 (7.8%)
Grade 12	403 (8.4%)
School location	
Urban	2691 (56.1%)
Rural	2110 (43.9%)
School workload	
Low	303 (6.3%)
Moderate	3841 (80.0%)
High	657 (13.7%)
Spherical equivalent of more myopic eye	
< −6.0 D	454 (9.5%)
-6.0 to <−0.5	3264 (68.0%)
-0.5 to 0.5 D	973 (20.3%)
> 0.5 D	110 (2.3%)
Uncorrected visual acuity in worse eye in vision	
Worse than 20/200	50 (1.0%)
20/200	314 (6.5%)
20/166	300 (6.3%)
20/133	400 (8.3%)
20/100	325 (6.8%)
20/80	351 (7.3%)
20/66	301 (6.3%)
20/50	290 (6.0%)
20/40	313 (6.5%)
20/33	180 (3.8%)
20/25	454 (9.5%)
20/20	1082 (22.5%)
20/16	328 (6.8%)
20/13	103 (2.1%)
20/10	10 (0.2%)
Wearing glasses	
No	2766 (57.6%)
Yes	2034 (42.4%)
Myopia: Spherical equivalent < −0.5D and visual acuity < 20/25 or worse	3030 (63.1%)
High myopia: Spherical equivalent < −6.0D and visual acuity 20/25 or worse	452 (9.4%)

Among all 4801 students, 2034 (42.4%) students wore glasses. However, among 3030 students with myopia, 1041 (34.5%) students did not wear glasses, 112 (10.8%) of them had UCVA 20/100 or worse, and 642 (62%) had UCVA 20/40 or worse (Table 2). Among 452 students with high myopia, 14 (3.1%) students did not wear glasses, half of them had UCVA 20/100 or worse and 79% of them had visual acuity 20/40 or worse (Table 2).

**Table 2:** The distribution of uncorrected visual acuity among myopia or high myopia yet not wear glasses

	Myopia yet not wearing glasses (*n* = 1041)	High myopia yet not wearing glasses (*n* = 14)
Uncorrected visual acuity in worse eye	n (%)	n (%)
< 20/200	2 (0.2%)	1 (7.1%)
20/200	13 (1.3%)	1 (7.1%)
20/166	16 (1.5%)	3 (21.4%)
20/133	42 (4.0%)	1 (7.1%)
20/100	39 (3.7%)	1 (7.1%)
20/80	74 (7.1%)	0 (0%)
20/66	109 (10.5%)	1 (7.1%)
20/50	140 (13.4%)	2 (14.3%)
20/40	207 (19.9%)	1 (7.1%)
20/33	124 (11.9%)	1 (7.1%)
20/25	275 (26.5%)	2 (14.3%)
Total	1041 (100%)	14 (100%)

**Fig. 1:**
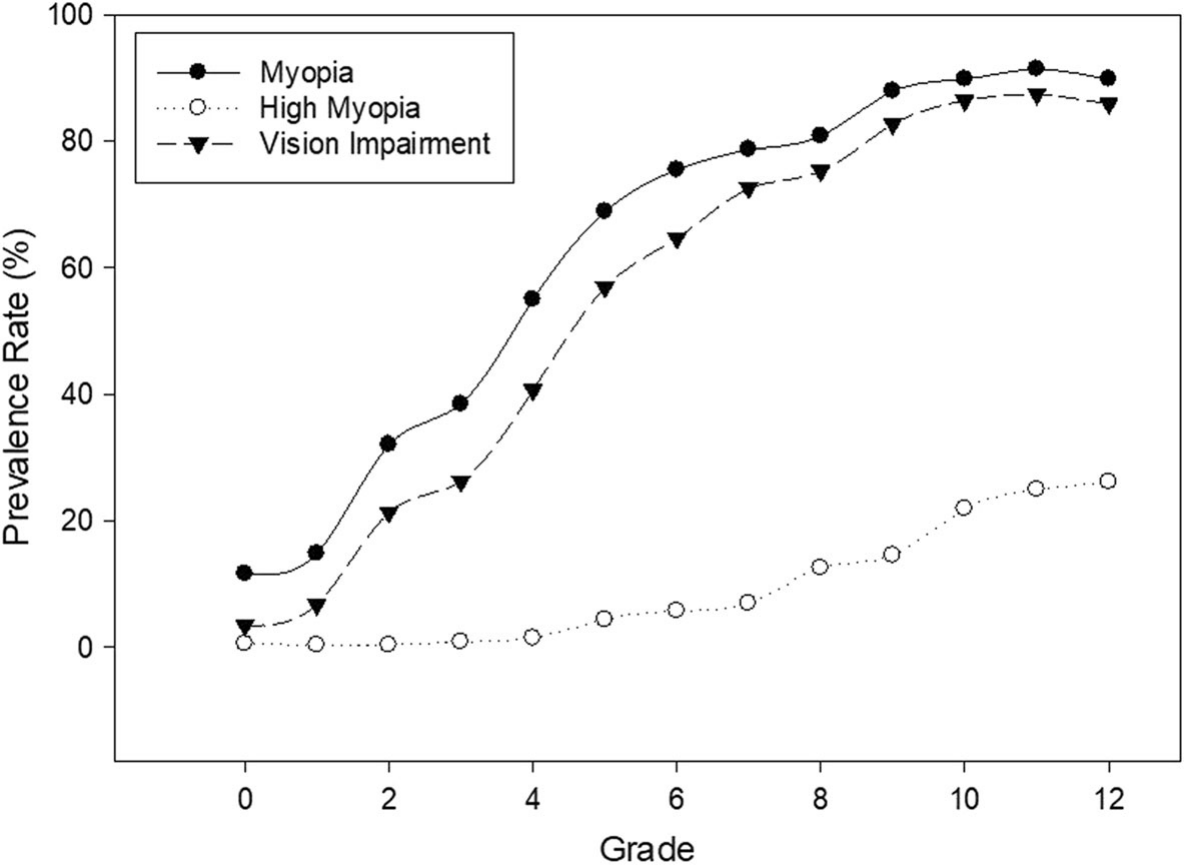
The prevalence rate of myopia, high myopia and vision impairment from kindergarten (grade 0) to grade 12. The figure shows the non-linear increase of myopia and vision impairment with increasing grade following the similar pattern

In univariate analysis (Table 3), increasing age and higher grade were significantly associated with more negative spherical equivalent, higher prevalence rates of myopia, high myopia and vision impairment (all *p* < 0.0001). The prevalence rate of myopia and vision impairment increased with grade following similar non-linear pattern (Fig. 1). The prevalence rates of myopia (and vision impairment) was 12% (3%) in kindergarten, 32% (21%) in grade 2, 69% (57%) in grade 5, 81% (75%) in grade 8 and approximately 90% (86%) by grade 10 or above. The prevalence rate of high myopia was relatively low in grade 4 or below (< 1.5%), 4–7% in grade 5 to 7, 13–15% in grade 8–9, and above 20% in grade 10–12 (Table 3).

**Table 3:** Refractive error and vision impairment by characteristics of students (*N* = 4801)

Demographics	N	Mean spherical equivalent (SD)	Myopia (%)	High myopia (%)	Vision impairment (%)
Age in years		*P* < 0.001	*P* < 0.0001	*P* < 0.0001	*P* < 0.0001
≤ 6	293	−0.23 (1.25)	35 (12.0%)	1 (0.3%)	9 (3.1%)
7	345	− 0.48 (1.29)	45 (13.0%)	2 (0.6%)	23 (6.7%)
8	359	−0.77 (1.24)	103 (28.7%)	1 (0.3%)	67 (18.7%)
9	369	−1.14 (1.55)	136 (36.9%)	3 (0.8%)	89 (24.1%)
10	360	−1.55 (1.39)	188 (52.2%)	3 (0.8%)	138 (38.3%)
11	385	−2.07 (1.74)	245 (63.6%)	13 (3.4%)	193 (50.1%)
12	353	− 2.61 (2.01)	267 (75.6%)	23 (6.5%)	222 (62.9%)
13	374	−2.94 (1.94)	290 (77.5%)	22 (5.9%)	270 (72.2%)
14	365	−3.24 (2.05)	298 (81.6%)	38 (10.4%)	280 (76.7%)
15	371	−3.72 (2.32)	317 (85.4%)	57 (15.4%)	292 (78.7%)
16	361	−4.16 (2.21)	326 (90.3%)	69 (19.1%)	313 (86.7%)
17	370	− 4.44 (2.53)	332 (89.7%)	88 (23.8%)	319 (86.2%)
18	370	−4.53 (2.38)	343 (92.7%)	96 (26.0%)	328 (88.7%)
≥ 19	126	−4.22 (2.92)	105 (83.3%)	36 (28.6%)	101 (80.2%)
Gender		*P* = 0.005	*P* < 0.0001	*P* = 0.68	*P* < 0.0001
Male	2647	−2.46 (2.47)	1572 (59.4%)	245 (9.3%)	1381 (52.2%)
Female	2154	−2.66 (2.36)	1458 (67.7%)	207 (9.6%)	1263 (58.6%)
Grade		*P* < 0.0001	*P* < 0.0001	*P* < 0.0001	*P* < 0.0001
Kindergarten	381	−0.24 (1.27)	44 (11.6%)	2 (0.5%)	13 (3.4%)
Grade 1	331	−0.52 (1.17)	49 (14.8%)	1 (0.3%)	22 (6.7%)
Grade 2	366	−0.83 (1.28)	117 (32.0%)	1 (0.3%)	78 (21.3%)
Grade 3	371	−1.22 (1.55)	143 (38.4%)	3 (0.8%)	97 (26.2%)
Grade 4	367	−1.66 (1.43)	202 (55.0%)	5 (1.4%)	149 (40.6%)
Grade 5	376	−2.32 (1.85)	259 (68.9%)	16 (4.3%)	214 (56.9%)
Grade 6	368	−2.62 (1.96)	278 (75.5%)	21 (5.7%)	238 (64.7%)
Grade 7	376	−2.97 (1.95)	296 (78.7%)	26 (6.9%)	273 (72.6%)
Grade 8	360	−3.37 (2.18)	291 (80.8%)	45 (12.5%)	271 (75.3%)
Grade 9	346	−3.79 (2.24)	304 (87.9%)	50 (14.5%)	286 (82.7%)
Grade 10	383	−4.29 (2.29)	344 (89.8%)	84 (21.9%)	331 (86.4%)
Grade 11	373	−4.50 (2.54)	341 (91.4%)	93 (24.9%)	326 (87.4%)
Grade 12	403	−4.45 (2.52)	362 (89.8%)	105 (26.1%)	346 (85.9%)
School location		*P* < 0.0001	*P* < 0.0001	*P* < 0.0001	*P* < 0.0001
Urban	2691	− 2.83 (2.49)	1797 (66.7%)	304 (11.3%)	1580 (58.7%)
Rural	2110	−2.18 (2.28)	1233 (58.4%)	148 (7.0%)	1064 (50.4%)
School workload		*P* < 0.0001	*P* < 0.0001	*P* < 0.0001	*P* < 0.0001
Low	303	−3.71 (2.21)	260 (85.8%)	45 (14.9%)	247 (81.5%)
Moderate	3841	−2.36 (2.37)	2310 (60.1%)	317 (8.3%)	1981 (51.6%)
High	657	−3.09 (2.58)	460 (70.0%)	90 (13.7%)	416 (63.3%)

Students in urban school had more negative refractive error (− 2.8 vs. -2.2 D, *p* < 0.001), higher prevalence rates of myopia (66.7% vs. 58.5%, *p* < 0.0001), high myopia (11.3% vs. 7.0%, *p* < 0.0001) and vision impairment (58.7% vs. 50.4%, *p* < 0.0001). Female students had more negative refractive error (− 2.7 D vs. -2.5 D, *p* = 0.006), higher prevalence rates of myopia (67.7% vs. 59.4%, *p* < 0.001) and vision impairment (58.6% vs. 52.2%, *p* < 0.0001), but similar in high myopia (9.6% vs. 9.3%, *p* = 0.68).

In multivariate analysis for the spherical equivalent (Table 4), higher grade (*p* < 0.0001), female (*p* = 0.02), urban school (*p* = 0.004), and high workload (*p* < 0.0001) were independently associated with more myopia refractive error (Table 4). These factors explained about 38% of the variation of refractive error across students. The grade alone explained 36% of the variation of refractive error across students.

**Table 4:** Multivariate analyses for the factors associated with spherical equivalent (*N* = 4804)

Demographics	N	Mean Spherical equivalent in diopter (SE)	*P*-value
Grade			< 0.0001
Kindergarten	381	0.03 (0.11)	
Grade 1	331	−0.23 (0.12)	
Grade 2	366	−0.54 (0.11)	
Grade 3	371	− 0.94 (0.11)	
Grade 4	367	−1.39 (0.12)	
Grade 5	376	−2.00 (0.11)	
Grade 6	368	− 2.32 (0.11)	
Grade 7	376	−2.71 (0.12)	
Grade 8	360	−3.11 (0.12)	
Grade 9	346	−3.52 (0.12)	
Grade 10	383	−4.24 (0.10)	
Grade 11	373	−4.41 (0.10)	
Grade 12	403	−4.31 (0.10)	
Gender			0.02
Male	2647	−2.22 (0.06)	
Female	2154	−2.35 (0.06)	
School location			0.004
Urban	2691	−2.37 (0.05)	
Rural	2110	−2.19 (0.07)	
School workload			< 0.0001
Low	303	−1.58 (0.13)	
Moderate	3841	−2.56 (0.03)	
High	657	−2.71 (0.09)	

Note: *R*^2^ = 0.378 from all these risk factors for predicting refractive error, which is higher than the *R*^2^ = 0.372 multivariate model using age instead of grade

In multivariate analysis for myopia (Table 5), higher grade (*p* < 0.0001), female (*p* < 0.0001) and higher workload (*p* = 0.007) were independently associated with higher prevalence rate of myopia, while school location (*p* = 0.63) was not associated with myopia prevalence. These factors predicted myopia with area under ROC curve (AUC) of 0.833 (95% CI: 0.821 to 0.845). Grade alone predicted myopia with AUC of 0.827 (95% CI: 0.814 to 0.839).

**Table 5:** Multivariate analyses for the factors associated with myopia and high myopia (N = 4804)

	Myopia		High Myopia	
Demographics	Odds ratio (95% CI)	*P*-value	Odds ratio (95% CI)	*P*-value
Grade		< 0.0001		< 0.0001
Kindergarten	Reference		Reference	
Grade 1	1.28 (0.83, 1.99)		0.57 (0.05, 6.34)	
Grade 2	3.43 (2.32, 5.06)		0.52 (0.05, 5.76)	
Grade 3	4.71 (3.22, 6.89)		1.55 (0.26, 9.33)	
Grade 4	9.42 (6.46, 13.7)		2.62 (0.51, 13.6)	
Grade 5	15.9 (10.8, 23.4)		8.40 (1.91, 36.9)	
Grade 6	23.2 (15.6, 34.5)		11.5 (2.67, 49.4)	
Grade 7	29.0 (19.4, 43.4)		14.1 (3.32, 59.7)	
Grade 8	33.1 (22.0, 50.1)		27.1 (6.52, 113)	
Grade 9	56.7 (36.1, 89.1)		32.1 (7.74, 133)	
Grade 10	80.1 (48.9, 131)		63.5 (15.4, 262)	
Grade 11	93.7 (56.2, 156)		73.2 (17.8, 301)	
Grade 12	78.4 (48.8, 126)		74.4 (18.1, 305)	
Gender		< 0.0001		0.76
Male	Reference		Reference	
Female	1.54 (1.33, 1.79)		1.03 (0.84, 1.27)	
School location		0.63		0.26
Urban	1.04 (0.89, 1.22)		1.15 (0.90, 1.47)	
Rural	Reference		Reference	
School workload		0.0007		< 0.0001
Low	Reference		Reference	
Moderate	1.88 (1.23, 2.86)		2.27 (1.55, 3.32)	
High	2.36 (1.52, 3.66)		2.28 (1.52, 3.41)	

Note: AUC = 0.833 from all these risk factors for predicting myopia, which is slightly higher than the AUC (0.832) from the multivariate using age instead of grade

AUC = 0.810 from all these risk factors for predicting myopia, which is higher than the AUC (0.809) from the multivariate using age instead of grade

In multivariate analysis of high myopia (Table 5), higher grade (*p* < 0.0001) and more intense education program (*p* < 0.0001) is independently associated with higher risk of high myopia, while gender (*p* = 0.76) and school location (*p* = 0.26) was not significantly associated with high myopia. These factors predicted high myopia with AUC of 0.810 (95% CI: 0.793 to 0.827). Grade alone predicted high myopia with AUC of 0.802 (95% CI: 0.785 to 0.819).

In multivariate analysis of vision impairment (Table 6), higher grade (*p* < 0.0001), female (*p* < 0.0001) and more intense education program (*p* < 0.0001) were independently associated with higher prevalence rate of vision impairment. School location (*p* = 0.28) was not significantly associated with vision impairment. These factors predicted vision impairment with AUC of 0.842 (95% CI: 0.831 to 0.854). Grade alone predicted vision impairment with AUC of 0.837 (95% CI: 0.825 to 0.848).

**Table 6:** Multivariate analyses for the factors associated with vision impairment (*N* = 4804)

Demographics	Odds ratio (95% CI)	*P*-value
Grade		< 0.0001
Kindergarten	Reference	
Grade 1	1.89 (0.94, 3.83)	
Grade 2	7.01 (3.81, 12.9)	
Grade 3	9.60 (5.26, 17.5)	
Grade 4	19.4 (10.8, 35.1)	
Grade 5	33.6 (18.6, 60.7)	
Grade 6	48.4 (26.7, 87.9)	
Grade 7	76.4 (42.0, 139)	
Grade 8	88.1 (48.2, 161)	
Grade 9	137 (73.9, 255)	
Grade 10	199 (104, 379)	
Grade 11	211 (110, 403)	
Grade 12	187 (99, 351)	
Gender		< 0.0001
Male	Reference	
Female	1.36 (1.18, 1.57)	
School location		0.28
Urban	0.92 (0.78, 1.08)	
Rural	Reference	
School workload		< 0.0001
Low	Reference	
Moderate	1.54 (1.06, 2.24)	
High	2.29 (1.54, 3.41)	

Note: AUC = 0.842 from all these risk factors for predicting vision impairment, which is slightly higher than the AUC (0.840) from the multivariate using age instead of grade

## Discussion

This study evaluated the prevalence of myopia, high myopia and vision impairment in school-aged students in the well-developed YiWu city of eastern China. We found that the overall rate among school-aged students was 63% for myopia, 9.4% for high myopia and 55.1% for vision impairment. These rates are similar to those reported in the school-aged children of well-developed East Asia countries including Singapore, Hongkong and Taiwan [15], but is higher than those reported in previous studies in China [10]. We found grade was associated with myopia and high myopia in a non-linear manner, with myopia prevalence rate of 12% even for kindergarten children, 76% for grade 6 elementary school students, above 80% for middle school students and approximately 90% for high school students. The prevalence rate of high myopia was low (0.5%) for kindergarten children, but it reached 5.7% for grade 6 elementary school students, 14.5% for grade 9 middle school students and 26% for grade 12 students. These high prevalence rates of myopia and high myopia suggests myopia is becoming a major public health problem in China.

Our study found that myopia is common even in kindergarten and increases in prevalence and severity through childhood. By grade 6 of elementary school, 76% students had myopia and 5.7% had high myopia. This prevalence rates of myopia and high myopia among elementary school students in this study were higher than previous studies [10, 15]. This high rate of myopia in elementary school students suggests that the myopia onset occurred earlier than before, likely due to earlier education than before in kindergarten and the increasing education burden on elementary school students. The intense education system leads to high number of hours on near work activities and reduced time on outdoor activities, which have been established as strong risk factors for myopia [6–9]. This earlier myopia onset can lead to high rate of high myopia by high school, as observed in our study that 24% high school students had high myopia. Since high myopia is associated with increased risk of vision loss and ocular diseases, prevention/intervention strategies should be developed to prevent myopia or its progression.

Compared to our study, the study by Pan et al. reported very low prevalence rate of myopia (2.4% in grade 1 and 29.4% in grade 7) and high myopia (0.1% in grade 1, 0.4% in grade 7) in 2346 rural Chinese school students with low education pressure in Mojiang of Southwestern China. However, their study also clearly showed the higher grade was associated with higher prevalence of both myopia and high myopia [5]. The low prevalence of myopia in school students in rural China suggest that Chinese students may not have a genetic predisposition to myopia and environmental factors may play a major role in the development of school myopia in Chinese children.

Our study found prevalence of myopia and high myopia was high in both elementary school students and high school students. A similarly high prevalence rate of myopia (76.5% in elementary school and 94.9% in junior high school) and high myopia (4.0% in elementary school, and 11.3% in junior high school) were recently reported in Japanese school students [16]. The high rate of myopia in both Japanese and Chinese students may be due to large amount of near work activities and limited time on outdoor activities, as students in China and Japan have high education pressure and are in the very competitive learning environment even among elementary school students.

Our study found the myopia prevalence rate increases with grade in a not linear manner, from prevalence rate of 11.6% in kindergarten, 14.8% in grade 1, jumped to 32% in grade 2, 55% by grade 4 and 69% by grade 5, and reached above 80% by grade 8 and 90% by grade 12. This increase in the prevalence rate of myopia can be partially due to the increase in the intensity of education system in China starting from elementary school [17]. However, previous studies in Chinese students found that the annual incidence of myopia is reasonably constant between ages of approximately 7 and 15 years, and by the age of 18 years, prevalence rate reached approximately 80% in urban-Chinese students regardless of the geographic locality [3, 10]. The large increase in the prevalence of myopia at 1 year after elementary school suggests that the optimal time for studying the effect of risk factors on myopia onset and for evaluating the prevention strategy for the onset of myopia.

Our study found that although myopia was common among school students, more than one third of myopic students did not wear glasses. Among those not wearing glasses, about 11% of them had very poor visual acuity of 20/100 or worse and more than half (60%) had visual acuity 20/40 or worse. These students needed glasses but they did not own glasses. Without correction by glasses for visual acuity, these students may not see the blackboard clearly, thus face challenges in effective learning in the classroom. A study of 7681 students aged 5 to16 years in rural Yunan of China found that among the students who needed glasses, only 18.9% owned them [18], which is lower than the study in urban (35.3%) [19] and rural (61.2%) China [20]. The reasons underlying low spectacles wearing among myopia might be multifactorial, including the lack of screening for detecting myopia, teasing or bullying by peers, the low attention and consciousness of the need for glasses, or the mis-conception by parents or students that wearing glasses will make the myopia worse [19–21]. A recent study found that the teasing or bullying by peers (48.6%) and the lost or forgot or stolen spectacles (26.1%) are the major reason of not wearing the prescribed glasses [21]. Thus, education on students and parents about the importance of timely screening for myopia and wearing glasses for best vision is very important to improve student learning and academic achievement.

Our study found that students in urban schools were likely immersed in an intensive education environment thus had higher prevalence rate of both myopia and high myopia. This is consistent with previous findings that location of residency (urban vs. rural) of an individual are associated with the likelihood of myopia [8], and the association between more near work or less outdoor activities are associated with higher risk of myopia [6, 7, 9].

The strength of the study includes the large sample size with representative sample of school students of all grades, allowing us to evaluate the association of myopia and vision impairment with age and grade. The study is limited in that the risk factors evaluated are very limited, and the refractive error was measured with non-cycloplegic autorefractor. Although our myopia definition was based on the combination of spherical equivalent and visual acuity to minimize the over-estimate myopia prevalence from using non-cycloplegic refractive error for defining the myopia, our estimate of myopia and high myopia can still be biased.

## Conclusions

This school-based cross-sectional study found that myopia and vision impairment are very common in school-age students, reaching alarming high in high school students. Increasing grade and higher school workload were associated with higher prevalence rate of myopia and vision impairment. Future longitudinal studied are needed to evaluate the progression from myopia to high myopia in school-aged children and to develop the intervention strategies to prevent or slow down the progression of myopia.

## Data Availability

De-identified data are available upon request to the first author.

## Abbreviations

AUC: Area under receiver operating curve
BCVA: Best corrected visual acuity
CI: Confidence interval
D: Diopter
SD: Standard deviation
SE: Standard error
SPHE: Spherical equivalent
